# Long-Term Observation of Higher-Order Aberrations and Microdistortions in Bowman’s Layer After Small Incision Lenticule Extraction for the Correcting Myopia With Spherical Equivalent Higher Than −9.0 Diopters

**DOI:** 10.3389/fmed.2022.814810

**Published:** 2022-04-08

**Authors:** Xueyi Zhou, Bing Qin, Tian Han, Jianmin Shang, Zhuoyi Chen, Jing Zhao, Peijun Yao, Xingtao Zhou

**Affiliations:** ^1^Department of Ophthalmology, Eye Institute, Eye and ENT Hospital, Fudan University, Shanghai, China; ^2^NHC Key Laboratory of Myopia, Key Laboratory of Myopia, Chinese Academy of Medical Sciences, Fudan University, Shanghai, China; ^3^Shanghai Research Center of Ophthalmology and Optometry, Shanghai, China; ^4^Shanghai Engineering Research Center of Laser and Autostereoscopic 3D for Vision Care (20DZ2255000), Shanghai, China

**Keywords:** high myopia, small incision lenticule extraction (SMILE), femtosecond laser-assisted *in situ* keratomileusis (FS-LASIK), higher-order aberrations, microdistortions in Bowman’s layer

## Abstract

**Purpose:**

To evaluate the outcomes in corneal higher-order aberrations (HOAs) and microdistortions in the Bowman’s layer after femtosecond laser small incision lenticule extraction (SMILE) for correcting extremely high myopia.

**Methods:**

This prospective study included patients with myopia with spherical equivalent ≥ -9.0 Diopters (D). SMILE was performed in forty eyes of 40 patients. Pentacam was used to evaluate HOAs before and at 1 day, 3 months, 6 months, and 2 years after surgery. Fourier-domain optical coherence tomography was used to evaluate microdistortions at 2 years postoperatively. Thirty-two eyes of 32 patients receiving femtosecond laser-assisted in situ keratomileusis (FS-LASIK) were enrolled as the control group. HOAs were measured before, at 1 day and at least 1 year postoperatively.

**Results:**

After SMILE, the long-term safety and effectiveness index was 1.25 and 0.85, respectively. Microdistortions were observed in 73.5% of the eyes at 2 years, with an average number of 1.20 ± 1.22 microdistortions and an average width of 287.37 ± 259.00 μm. We detected more microdistortions in the horizontal meridian than in the vertical meridian (*p* = 0.035). The average number and width of microdistortions were both higher in the central region (≤4 mm) than in the peripheral region (4–8 mm) (both *p* < 0.001). With the exception of horizontal trefoil in the SMILE group and vertical trefoil in the FS-LASIK group, significant changes over time were observed in all other HOAs (all *p* < 0.05). Meanwhile, we detected significant increases in the total corneal HOA, spherical aberration (SA), and coma at all time-points after both surgeries (all *p* < 0.01). Compared with FS-LASIK, SMILE induced less SA (*p* < 0.001) and more horizontal coma (*p* = 0.036). In the SMILE group, the HOA, SA, and trefoil were more in the small optical zone (≤6.0 mm) than in the large optical zone (>6.0 mm) (all *p* < 0.05). The increase in SA and most trefoil correlated with the mean number of central microdistortions number (all *p* < 0.05).

**Conclusion:**

For myopia over −9.0D, the microdistortions in the Bowman’s layer were still detectable in most eyes long-term after SMILE. Both SMILE and FS-LASIK induced more HOAs, mainly HOA, SA, and coma. The small optical zone and microdistortions may affect postoperative aberrations.

## Introduction

Femtosecond laser small incision lenticule extraction (SMILE) has been widely used since it was first introduced in 2011 (1), and its long-term safety, predictability, efficacy, and stability have been confirmed (2). A major shortcoming of SMILE surgery is that it is not possible to perform individualized treatments guided by topographic maps or wavefronts (3). Studies have pointed out that corneal refractive surgeries can induce a significant increase in corneal higher-order aberrations (HOAs) (4–6). The increase in aberrations may degrade postoperative visual quality and result in night vision disturbances, halos, glare, ghosting, and monocular diplopia (7). Notably, the increase in corneal HOAs was reported to be higher in patients with high myopia (8, 9). Moreover, other factors, such as pupil size or optical zone (OZ), also exert great influence on corneal HOAs (7).

Bowman’s layer is a non-renewable acellular structure between the epithelium and stroma and has been proven to be the strongest part of the cornea (10). Compared with traditional laser-assisted in situ keratomileusis (LASIK), the flapless SMILE procedure retains a stronger anterior corneal stroma and minimizes disruption to the Bowman’s layer. It has been demonstrated that SMILE has more advantages in corneal biomechanics than LASIK (11, 12). Although flap-related complications are avoided with this procedure, SMILE introduces a new problem. Our research team reported of microdistortions for the first time in 2013 that the Bowman’s layer lost its original smooth structure and showed segmental distortion after SMILE (13). According to previous studies, microdistortions have little effect on postoperative aberrations or optical quality (5, 13). However, it is important to note that these microdistortions can persist for up to 3 years postoperatively in patients with high myopia, and the risk increases with the increase in corrected diopters (D) or refractive lenticule thickness (14, 15).

At present, the SMILE surgery is able to correct myopia and myopic astigmatism over −10 D (16). However, there are currently limited published reports that delve into the SMILE procedure’s long-term effects in these populations, and a direct comparison between the long-term postoperative change of SMILE and LASIK is lacking from the literature. This study aimed to report the Bowman’s layer microdistortions that arise at 2 years after SMILE and compare the long-term changes in corneal HOAs after SMILE and femto-second (FS)-LASIK for correcting myopia with spherical equivalent (SE) higher than -9.0 D.

## Materials and Methods

### Patient Population

Patients were consecutively recruited between May 2015 and August 2016 at EENT Hospital of Fudan University (Shanghai, China). Inclusion criteria were age ≥ 18 years old, SE ≥ -9.0 D, astigmatism < 5.0 D, a stable refractive error over the last 2 years (change ≤ 0.50 D), transparent cornea, normal corneal topography, and no contact lens wear at least 2 weeks preoperatively. Exclusion criteria included insufficient corneal thickness [central corneal thickness < 450 μm or estimated postoperative corneal residual bed thickness (RBT) < 250 μm]; keratoconus or suspected keratoconus; active eye infection; severe dry eye; and presence or history of other ocular (e.g., cataract, glaucoma, retinopathy, ocular trauma, or surgery) or systemic diseases. All participants were screened routinely and met surgical indications. Baseline and surgical information are shown in Table 1.

**TABLE 1:** Baseline and surgical information of small incision lenticule extraction (SMILE) and femtosecond laser-assisted *in situ* keratomileusis (FS-LASIK).

Parameters	SMILE (*N* = 40)	FS-LASIK (*N* = 32)	*P*-value
Age (years)	30.30 ± 9.21 (20–55)	34.13 ± 13.04 (20–63)	0.395
Sphere (D)	−9.83 ± 1.30 (−7.75 ∼−12.75)	−10.48 ± 1.21 (−8.25 ∼−13.25)	0.026
Cylinder (D)	−1.34 ± 0.92 (−0.25 ∼−4.50)	−1.34 ± 0.94 (0 ∼−3.50)	0.973
SE (D)	−10.50 ± 1.15 (−9.00 ∼−13.88)	−11.15 ± 1.09 (−9.00 ∼−14.50)	0.003
UDVA	0.03 ± 0.04 (0.01–0.1)	0.02 ± 0.02 (0.01–0.1)	0.229
CDVA	1.00 ± 0.12 (0.7–1.2)	0.92 ± 0.19 (0.9–1.2)	0.064
TCT (μm)	550.05 ± 26.87 (503–628)	559.25 ± 36.02 (490–620)	0.234
IOP (mmHg)	16.41 ± 2.40 (10–22)	16.01 ± 3.21 (9–21)	0.545
Dark pupil (mm)	6.78 ± 0.81 (5.0∼8.1)	6.75 ± 0.70 (4.8∼8.1)	0.742
Axial lengths (mm)	27.49 ± 0.95 (26.02∼29.69)	27.78 ± 0.92 (26.14∼29.17)	0.202
Optical zone (mm)	6.15 ± 0.24 (5.7∼6.5)	5.97 ± 0.21 (5.75∼6.5)	0.001
Ablation depth (μm)	150.90 ± 10.18 (105∼164)	148.53 ± 10.33 (115∼159)	0.222
RBT (μm)	283.65 ± 22.42 (257–349)	310.72 ± 32.90 (261–366)	0.000

*D, diopters; SE, spherical equivalent; UDVA, uncorrected distance visual acuity; CDVA, corrected distance visual acuity; TCT, thinnest corneal thickness; IOP, intraocular pressure; RBT, residual bed thickness. Data are described as mean ± standard deviation (range).*

Forty participants (12 male and 28 female) who received SMILE were examined before and at 1 day, 3 months, 6 months, and 2 years postoperatively. Thirty-two participants (9 male and 23 female) who underwent FS-LASIK were enrolled as the control group, and were examined before and at 1 day and long-term (mean: 17.22 ± 4.43 months, range from 12 to 30 months) postoperatively. The study adhered to the tenets of the Declaration of Helsinki and was approved by the Ethics Committee of Eye and ENT Hospital of Fudan University. All participants were fully informed and signed written consent.

### Surgical Procedure

All surgeries were performed by a highly experienced doctor (XTZ). The details of both procedures have been reported previously (1, 2).

We used a VisuMax femtosecond laser (Zeiss Medical Technology, Jena, Germany) to perform the SMILE procedure. The femtosecond laser scans were set as follows: repetition rate, 500 kHz; pulse energy, 130 nJ, targeted lenticule diameter, 5.7–6.5 mm, and intended cap thickness, 100–120 μm. The cap diameter was 1 mm larger than that of the lenticule. The side cut was set at the 12:00 clock position with a width of 2 mm.

In FS-LASIK procedure, flap was created with the 500 kHz VisuMax femtosecond laser system and stromal tissue ablation was performed with a 250 kHz MEL 80 excimer laser (Zeiss Medical Technology, Oberkochen, Germany). The pulse energy of femtosecond laser was set to 185 nJ. The flap thickness and flap diameter were set to 100 μm and 8 mm, respectively. The hinges were located in a superior orientation with a length of 4.0 mm.

A therapeutic soft contact lens (ACUVE OASYS Inc., Jacksonville, FL, United States) was used for 1 day after FS-LASIK. Artificial tears, levofloxacin (0.5%), and fluorometholone (0.1%) were routinely used after both procedures. The postoperative treatment protocol has been previously described (2).

### Microdistortion Measurement

Anterior segment Fourier-domain optical coherence tomography (FD-OCT, RTVue, software version 6.2; Optovue, Inc., Fremont, CA) was used to observe the morphological features of the Bowman’s layer. Linear scanning along the four meridians (0°, 45°, 90°, and 135°) was performed for each eye. On the FD-OCT image, the normal Bowman’s layer appears as a smooth double-line structure, and the twisted segments are microdistortions, which have been defined previously (13). The total number and width of microdistortions on the four meridians were counted and used to calculate the mean number and width per meridian.

### Aberration Measurement

The Pentacam HR (oculus GmbH, Wetzlar, Germany) was used to measure corneal aberrations before and after surgery. All measurements were performed in a standard, dark environment. Participants were instructed to fixate on a light inside the rotating camera after blinking freely, and the scan was taken immediately. At least three consecutive scans were performed for each eye, and only scans whose quality was labeled as “OK” were selected and analyzed. Root mean square (RMS) values were calculated using Zernike polynomials, including total corneal HOA, horizontal coma $\left(Z_{3}^{1}\right)$, vertical coma ($Z_{3}^{-1}$), horizontal trefoil ($Z_{3}^{3}$), vertical trefoil ($Z_{3}^{-3}$), spherical aberration (SA, $Z_{4}^{0}$), 3rd order coma (coma3), 3rd and 5th order coma (coma5), 3rd order trefoil (trefoil3), and 3rd and 5th order trefoil (trefoil5). Coma3, coma5, trefoil3 and trefoil5 was calculated by $\sqrt{{\left(Z_{3}^{1}\right)}^{2}+{\left(Z_{3}^{-1}\right)}^{2}}$,$\sqrt{{\left(Z_{3}^{3}\right)}^{2}+{\left(Z_{3}^{-3}\right)}^{2}}$, $\sqrt{{\left(Z_{3}^{1}\right)}^{2}+{\left(Z_{3}^{-1}\right)}^{2}+{\left(Z_{5}^{1}\right)}^{2}+{\left(Z_{5}^{-1}\right)}^{2}}\,\sqrt{{\left(Z_{3}^{3}\right)}^{2}+{\left(Z_{3}^{-3}\right)}^{2}+{\left(Z_{5}^{3}\right)}^{2}+{\left(Z_{5}^{-3}\right)}^{2}}$, respectively. In addition, we grouped participants into low and high OZ groups (OZ ≤ preset value and OZ > preset value, respectively), with preset values of 5.8, 6.0, 6.2, and 6.4 mm; aberrations were compared between the two groups in each set.

### Statistical Analysis

Data were analyzed using the SPSS v26.0 software package (SPSS, Inc., IL, United States). One eye from each patient was randomly selected for analysis. Normality was checked using the one-sample Kolmogorov-Smirnov test. Categorical variables were reported as frequency and percentage, and mean ± standard deviation (SD) or mean ± standard error (SE) was used for continuous variables. The Wilcoxon signed-rank test was used to compare data of microdistortions in different areas. The long-term variations of aberrations were compared between the low and high OZ groups using independent sample *t*-tests or Mann-Whitney *U*-tests. A mixed linear model for repeated measurements was used for pre- and post-operative comparison, and comparison between different surgeries or OZ groups. Pearson or Spearman correlation analyses was used for correlation analysis. Statistical significance was defined as *p* < 0.05.

## Results

### Visual Outcomes

All surgeries were performed without complications. At 2 years after SMILE, the safety and efficacy indices were 1.25 and 0.85, respectively. The uncorrected distance visual acuity (UDVA) was ≥ 20/32, ≥ 20/25, and ≥ 20/20 in 32 (94.1%), 24 (70.6%), and 15 (44.1%) eyes, respectively. The SE of 35.3% and 52.9% eyes were within ± 0.50 D and ± 1.00 D, respectively. All eyes had a corrected distance visual acuity (CDVA) equal to or better than the preoperative value, and 41.2% (14/34) of the eyes gained 1–3 lines of CDVA; no eye lost lines.

### Microdistortions in Bowman’s Layer

Bowman’s layer microdistortion was observed in 73.5% eyes at 2 years after SMILE, with an average microdistortion number of 1.20 ± 1.22 per meridian and an average microdistortion width of 287.37 ± 259.00 μm per meridian. We detected more microdistortions in the horizontal meridian than in the vertical meridian (1.59 ± 1.56 vs. 1.03 ± 1.53, *Z* = -2.105, *p* = 0.035), while no significant difference was observed in the microdistortion width (*Z* = -1.521, *p* = 0.128). No significant difference was detected between the nasal-temporal and inferior-superior areas. The average number (1.18 ± 1.20 vs. 0.02 ± 0.07, *Z* = -4.378, *p* < 0.001) and width (281.72 ± 252.23 μm vs. 5.65 ± 18.71μm, *Z* = -4.372, *p* < 0.001) of microdistortions were both higher in the central region (≤4 mm) than in the peripheral region (4–8 mm).

### Aberration Outcomes

**FIGURE 1:**
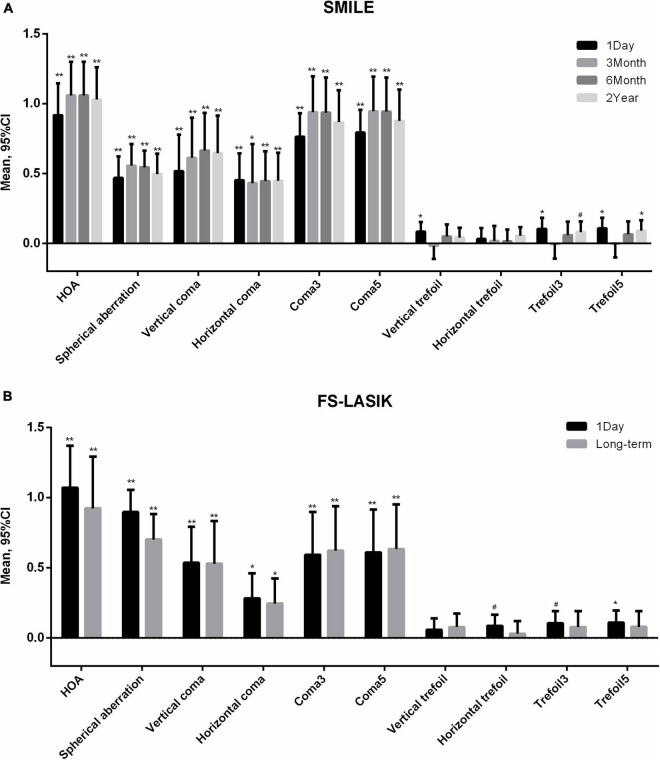
Variations of higher-order aberrations after **(A)** small incision lenticule extraction (SMILE) and **(B)** femtosecond laser-assisted in situ keratomileusis (FS-LASIK). Compared with baseline **p < 0.001, *p < 0.01, ^#^*p* < 0.05. HOA, total higher-order aberration; Coma3, 3rd order coma; Coma5, 3rd and 5th order coma; Trefoil3, 3rd order trefoil; Trefoil5, 3rd and 5th order trefoil.

Table 2 shows the aberrations before and after surgery. With the exception of horizontal trefoil in the SMILE group and vertical trefoil in the FS-LASIK group, significant changes over time were observed in all aberration parameters. Figure 1 shows the variations in aberration at different follow-up points. Compared with baseline, there were significant increases in all aberration parameters, except trefoil, at all postoperative time-points for both surgeries (all *p* < 0.01). In the SMILE group, trefoil3 and trefoil5 increased significantly at 1 day (*p* = 0.003, 0.001, respectively) and 2 years (*p* = 0.041, 0.008, respectively) postoperatively, while vertical trefoil only increased significantly at 1 day (*p* = 0.007). In the FS-LASIK group, horizontal trefoil (*p* = 0.033), trefoil3 (*p* = 0.013), and trefoil5 (*p* = 0.008) only increased significantly at 1 day. Most aberrations with significant changes, including HOA, SA, coma3, and coma5, reached a peak 3 months after SMILE and then decreased gradually. Horizontal coma and all trefoil aberrations reached a peak at 1 day, and gradually increased after a short decline at 3 months after surgery. Only the vertical coma reached a peak at 6 months. In the FS-LASIK group, most aberrations reached a peak at 1 day postoperatively, except for coma3 and coma5.

**TABLE 2:** Higher-order aberrations before and after small-incision lenticule extraction (SMILE) and femtosecond laser-assisted in situ keratomileusis (FS-LASIK).

	SMILE					FS-LASIK				
Parameter	Pre-op	1 Day	3 month	6 month	Long-term	*P*-value	Pre-op	1 Day	Long-term	*P*-value
HOA	0.386 ± 0.021	1.303 ± 0.076**	1.444 ± 0.080**	1.437 ± 0.083**	1.415 ± 0.075**	0.000	0.466 ± 0.053	1.536 ± 0.114**	1.388 ± 0.120**	0.000
Spherical aberration	0.186 ± 0.013	0.655 ± 0.052**	0.741 ± 0.052**	0.730 ± 0.041**	0.683 ± 0.049**	0.000	0.215 ± 0.029	1.112 ± 0.071**	0.917 ± 0.054**	0.000
Vertical coma	0.185 ± 0.021	0.701 ± 0.083**	0.797 ± 0.089**	0.849 ± 0.084**	0.830 ± 0.080**	0.007	0.194 ± 0.035	0.729 ± 0.106**	0.723 ± 0.114**	0.000
Horizontal coma	0.080 ± 0.009	0.531 ± 0.063**	0.511 ± 0.087*	0.525 ± 0.072**	0.527 ± 0.068**	0.000	0.106 ± 0.054	0.387 ± 0.062*	0.350 ± 0.054*	0.000
Coma3	0.214 ± 0.063	0.976 ± 0.069**	1.152 ± 0.087**	1.149 ± 0.078**	1.081 ± 0.063**	0.000	0.237 ± 0.042	0.830 ± 0.124**	0.858 ± 0.115**	0.000
Coma5	0.223 ± 0.062	1.015 ± 0.068**	1.167 ± 0.085**	1.165 ± 0.077**	1.098 ± 0.063**	0.000	0.249 ± 0.043	0.859 ± 0.124**	0.883 ± 0.114**	0.000
Vertical trefoil	0.18 ± 0.017	0.191 ± 0.021*	0.090 ± 0.029	0.157 ± 0.025	0.149 ± 0.018	0.002	0.130 ± 0.018	0.188 ± 0.028	0.207 ± 0.035	0.072
Horizontal trefoil	0.088 ± 0.010	0.118 ± 0.024	0.104 ± 0.032	0.101 ± 0.142	0.142 ± 0.018	0.145	0.110 ± 0.023	0.195 ± 0.022^#^	0.139 ± 0.024	0.028
Trefoil3	0.150 ± 0.019	0.254 ± 0.023*	0.146 ± 0.032	0.209 ± 0.027	0.229 ± 0.019^#^	0.000	0.191 ± 0.025	0.295 ± 0.026^#^	0.268 ± 0.038	0.014
Trefoil5	0.157 ± 0.018	0.265 ± 0.022*	0.156 ± 0.031	0.221 ± 0.026	0.247 ± 0.019*	0.000	0.198 ± 0.024	0.308 ± 0.026*	0.277 ± 0.038	0.009

HOA, total higher-order aberration; Coma3, 3rd order coma; Coma5, 3rd and 5th order coma; Trefoil3, 3rd order trefoil; Trefoil5, 3rd and 5th order trefoil. Data are described as mean ± standard error. Multiple significance tests were conducted by the Bonferroni method (Compared with pre-operation **P < 0.001, *P < 0.01, ^#^P < 0.05).

Significant differences were observed in SA (*p* < 0.001) and horizontal coma (*p* = 0.036) between two surgeries. No significant preoperative differences were observed. Compared with the SMILE group, the SA was significantly higher in the FS-LASIK group at 1 day (difference: 0.471 ± 0.081, *p* < 0.001) and in the long-term (difference: 0.227 ± 0.073, *p* = 0.003), and horizontal coma was significantly lower at 1 day (difference: −0.184 ± 0.085, *p* = 0.032) and in the long-term (difference: −0.176 ± 0.074, *p* = 0.018).

### Aberration Changes of Different Optical Zone

**FIGURE 2:**
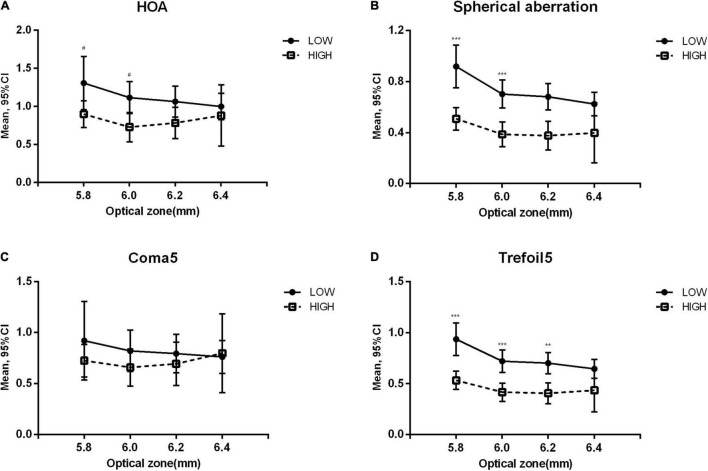
Variation of higher-order aberrations at the last follow-up in low and high optical zone groups. Low: optical zone less than or equal to the value on the corresponding horizontal axis. High: optical zone higher than the value on the corresponding horizontal axis. Compared between optical zone groups: ***p < 0.001, **p < 0.01, ^#^*p* < 0.05. **(A)** Total higher-order aberration (HOA). **(B)** Spherical aberration. **(C)** 3rd and 5th order coma (Coma5). **(D)** 3rd and 5th order trefoil (Trefoil5).

Figure 2 shows the long-term increase in aberrations in the high and low OZ groups. As the abscissa value increased from 5.8 to 6.4 mm, the difference between high and low OZ groups decreased gradually. HOA, SA, and trefoil5 were significantly higher in the OZ ≤ 5.8 mm group than in the OZ > 5.8 mm group (*p* = 0.032, < 0.001, < 0.001, respectively) and in the OZ ≤ 6.0 mm group than in the OZ > 6.0 mm group (*p* = 0.019, < 0.001, < 0.001, respectively). However, a significant difference in trefoil5 (*p* = 0.001) was only detected between the OZ ≤ 6.2 mm group and the OZ > 6.2 mm group. No significant difference was observed between the OZ ≤ 6.4 mm group and the OZ > 6.4 mm group.

**FIGURE 3:**
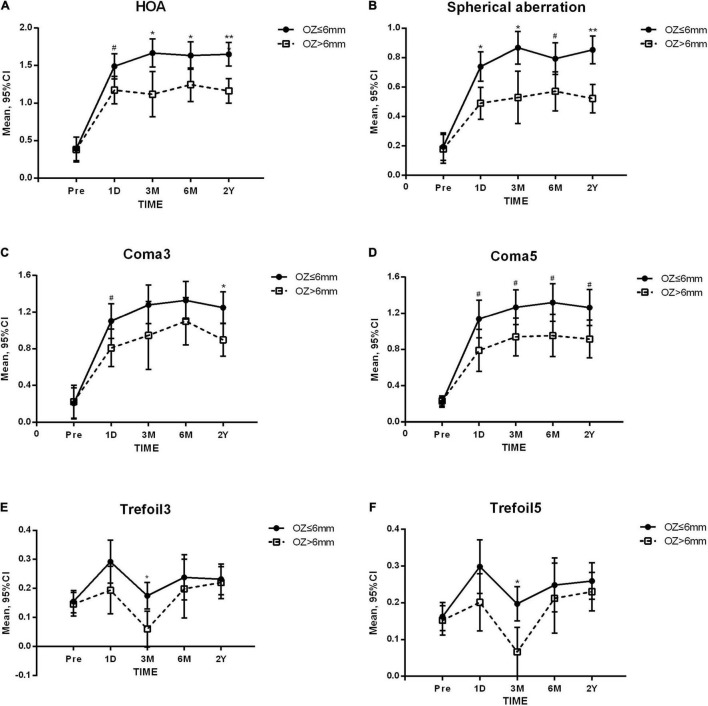
Higher-order aberrations before and after small incision lenticule extraction (SMILE). OZ, optical zone. Compared between OZ groups: ** *p* < 0.001 * *p* < 0.01 ^#^*p* < 0.05, **(A)** Total higher-order aberration (HOA). **(B)** Spherical aberration. **(C)** 3rd order coma (Coma3). **(D)** 3rd and 5th order coma (Coma5). **(E)** 3rd trefoil (Trefoil3). **(F)** 3rd and 5th order trefoil (Trefoil5).

We further investigated the results at all time-points when grouped by 6.0 mm. In the SMILE group, the increases in a large variety of aberrations, including HOA (*p* = 0.005), SA (*p* = 0.001), coma3 (*p* = 0.047), coma5 (*p* = 0.016), trefoil3 (*p* = 0.021), and trefoil5 (*p* = 0.015), in the low OZ group were significantly higher than those in the high OZ group. Significant differences were detected at all postoperative time-points for HOA, SA, and coma5, at 1 day and long-term for coma3, and at 3 months for trefoil3 and trefoil5 (Figure 3). However, there was no significant difference between the low and high OZ groups in the FS-LASIK group.

When comparing SMILE and FS-LASIK, significant differences were observed in SA (*p* < 0.001) and coma5 (*p* = 0.022). In the low OZ group, the SA was significantly higher in the FS-LASIK group than in the SMILE group at 1 day (difference: 0.332 ± 0.078, *p* < 0.001), while in the high OZ group, significant differences were observed at both 1 day (difference: 0.635 ± 0.235, *p* = 0.008) and in the long-term (difference: 0.257 ± 0.118, *p* = 0.031). The coma5 of FS-LASIK was significantly lower than that of SMILE in the low OZ group (long-term difference: −0.350 ± 0.160, *p* = 0.032), but not in the high OZ group.

### Correlation Analysis

In the SMILE group, the mean number of central microdistortions negatively correlated with corrected cylindrical refraction (*r* = -0.476, *p* = 0.004), SE (*r* = -0.363, *p* = 0.035), OZ (*r* = -0.548, *p* = 0.001) and RBT (*r* = -0.374, *p* = 0.030), but these microdistortions were not associated with either UDVA (*p* = 0.718) or CDVA (*p* = 0.213) at 2 years.

The increase in SA correlated with the preoperative SE (*r* = -0.596, *p* = 0.000) and spherical refraction (*r* = -0.587, *p* = 0.000). The increase in HOA (*r* = -0.410, *p* = 0.016), SA (*r* = -0.489, *p* = 0.003), vertical trefoil (*r* = -0.341, *p* = 0.048), and horizontal trefoil (*r* = -0.479, *p* = 0.004) correlated with OZ. In addition, the increase in various aberration parameters correlated with the mean number of central microdistortions, including SA (*r* = 0.383, *p* = 0.025), horizontal trefoil (*r* = 0.375, *p* = 0.029), vertical trefoil (*r* = 0.369, *p* = 0.032), trefoil3 (*r* = 0.344, *p* = 0.046), and trefoil5 (*r* = 0.361, *p* = 0.036).

## Discussion

In this study, Bowman’s layer microdistortion existed in 73.5% eyes at 2 years following SMILE, which seems higher than the results observed in high myopia of less than −9.0 D (65.4–68.42%) (5, 14). This finding is in agreement with the previous concept that patients with higher myopia are more likely to develop more microdistortions. It has been reported that in LASIK procedure, the posterior surface area of corneal flap is larger than the residual stromal bed, and that the excess area may form a potential overlap at the edge (17). In SMILE, however, the curvature of posterior surface of the corneal cap is steeper than the residual stromal bed, and this disparity cannot be accommodated by flap overlap, which may partly explain why microdistortion seems more common after SMILE. The occurrence of microdistortions has been reported to be correlated with refraction degree, corneal thickness, lenticule thickness, and corneal curvature (5, 13, 14). Similarly, this study shows that higher preoperative refraction, thinner RBT, and smaller OZ were associated with more microdistortions. One possible reason is that a higher magnitude of myopia correction may result in a larger difference in curvature between interfaces after lenticule extraction, and that this mismatch may be accommodated by fractional compression instead of flap overlap. Small OZ leaves limited space for morphological adaptation. In addition, biomechanical weakening of the cornea indicates less resistance to corneal deformation (18). We suspect that the correction of more severe myopia, accompanied by thicker lenticule and thinner RBT, makes it more difficult to retain corneal biomechanics or maintain corneal shape. In this study, the center region presented more and wider microdistortions than the peripheral region, which is consistent with previous studies (13, 14). Additionally, the horizontal meridian presented more microdistortions than the vertical meridian. There is some evidence that corneal thickness is less in the horizontal than in the vertical direction (19); this finding might partly explain the above outcome we observed, considering that a thinner cornea is more likely to induce microdistortions (5).

In previous studies, the microdistortions after SMILE were not associated with visual acuity or intraocular scattering and were only weakly associated with the increase of fifth-and sixth-order aberrations (5, 13, 15, 16). Therefore, researchers have speculated that Bowman’s layer microdistortions have little effect on optical quality. We found that the number of microdistortions was not significantly associated with long-term visual acuity, which is consistent with previous results, but it was significantly correlated with the induction of SA and various trefoils. This discrepancy could be attributed to the higher severity of myopia in this research. Both SA and trefoil were considered as visually significant HOAs, while other aberrations above the fourth-order were relatively less significant (20). Therefore, the impact of microdistortions on the long-term visual quality of extremely high myopia needs to be further investigated.

The present study showed that aberrations increased significantly after SMILE and FS-LASIK procedures, mainly for HOA, SA, and coma. This is in agreement with previous reports, which revealed that SA and coma were the main causes of induced HOA after SMILE and FS-LASIK (2, 5, 6). Moreover, the postoperative SA increased with the increase in preoperative SE and spherical refraction, which is in agreement with the findings of a previous study (8). According to Pedersen et al. SA and HOA decreased significantly from 3 months to 3 years after SMILE, while coma remained stable (21). Li et al. found that the HOA, SA, and coma reached a peak 3–6 months after SMILE (2). In this study, HOA, SA, and coma all reached a peak at 3 months after SMILE and then decreased gradually, but not significantly. We suspect that the greatest increase in aberration may occur earlier in eyes with extremely high myopia and may decrease more slowly over the long term. Furthermore, reports on significant increases in postoperative trefoils are limited. In this study, a significant increase in trefoil was observed following SMILE. Interestingly, we found a slight decrease in trefoil 3 months postoperatively, followed by a progressive increase since then a significant increase was also noticed at 2 years compared with baseline, which is worthy of further observation.

In line with the long-term findings of the meta-analysis, we also found that FS-LASIK procedure induced more SA than SMILE procedure (22). One possible explanation is that the flap and stromal bed had a significant effect on the induced aberrations after corneal refractive surgery. Eyes that underwent FS-LASIK carry a potential risk of corneal flap deflection or displacement. For safety concerns, relatively thin corneal flaps might be designed in patients with high myopia, which may further increase this risk, whereas the flapless characteristic of the SMILE procedure eliminates this risk (22). Moreover, SMILE resulted in less reduction of the effective optical zone than FS-LASIK after correction (23), which might also partly explain the difference in SA. However, we also found that the long-term horizontal coma increased more in the SMILE group, consistent with a previous report (2). Imbalanced corneal healing response or optical changes might be responsible for the induction of postoperative coma (24). Ablation decentration is a common complication after refractive surgery, and has a significant impact on HOA, particularly coma (25, 26). The SMILE procedure currently lacks an eye-tracking system and relies more on physician experience and patient cooperation, which may increase the risk of decentration.

It is well known that larger OZs are more likely to obtain better postoperative visual quality and less HOA, but more corneal stroma will be cut. For extremely high myopia, in order to achieve refraction correction and meanwhile ensure surgical safety as much as possible, surgeons usually prefer to reduce the OZ during correction to preserve more corneal tissue. However, these patients are at a higher risk of halos, glare, reduced contrast sensitivity, and refractive regression after surgery (27). In the current study, we noticed that it is quite common to obtain a relatively smaller OZ than dark pupil for extremely high myopia, which occurred in 70% of patients in the SMILE group and 81.3% in the FS-LASIK group. In addition, correlation analysis revealed that small OZ was significantly correlated with HOA, SA, and coma. While it is difficult to ensure sufficient OZ, it is important to understand the influence of a small OZ on aberrations. Therefore, we grouped participants by different OZ values from high to low and found that its impact on aberrations became more apparent as OZ decreased, particularly when the OZ was equal to or less than 6 mm, mainly affecting HOA and SA. It is worth noting that SMILE was more affected by a small OZ. Intended OZ greater than 6 mm can significantly improve HOA, SA, coma, and trefoil after SMILE (Figure 3). When comparing between surgeries, it was found that SMILE induced less SA than FS-LASIK, and that this advantage grew when the OZ was greater than 6 mm. In addition, a higher induction of coma in the SMILE group was only observed in eyes with low OZ (≤ 6 mm). These results suggest that an intended OZ greater than 6 mm for the SMILE procedure may optimize postoperative visual quality.

As mentioned above, it is worth mentioning that keratorefractive surgeries have their own limitations when applied to cases of extremely high myopia, which may result in increased postoperative HOA and poor visual quality. Moreover, prolonged visual recovery, refraction regression, and weak biomechanical property are also concerning. Too much corneal tissue removed and a relatively thinner residual stroma bed could also increase the risk of post-laser corneal ectasia (28). Implantable collamer lens (ICL) implantation is another widely used and well-known therapy for correcting high myopia, and the range of the target population has broadened since it was first introduced (29). Moshirfar et al. found SMILE may be comparable to ICL for high myopia correction on postoperative SE controls (30), in agreement with the results of Cao et al. (29) and Niu et al. (31). In addition, ICL showed better performance on efficacy and safety indices and induced less HOAs (29, 32). From this standpoint, ICL might be a better option for patients with high myopia. However, we also note that safety is one of the major concerns for ICL implantation. There might be potential complications of long-term endothelial cell loss, glaucoma, and cataract formation (28). For patients who would be poor candidates for ICL, such as those with a shallow anterior chamber, relatively low endothelial cell count, or high intraocular pressure, or who were unable to maintain long-term regular postoperative follow-up or have a strong desire for keratorefractive surgeries, SMILE and FS-LASIK are alternative choices, but impaired optical quality should be informed in advance, especially with small optical zone. Overall, we would not recommend corneal refractive surgeries for high myopia population with small OZ, clinicians have to weigh the pros and cons and make the proper choice for extremely high myopia.

This study had some limitations. First, we only evaluated the change in objective indicators, such as aberrations and microdistortions, and did not investigate subjective visual quality indicators. In addition, due to the very limited number of patients with myopia over −9.0 D for corneal refractive surgeries, the sample size was relatively small in this study. We expect to conduct a study with a larger sample size or a comparison with moderate to high myopia in the future. Third, we only measured the microdistortion outcomes after SMILE and did not compare the results with FS-LASIK; future comparisons are expected. Finally, we measured the aberrations at 1 day after removing the contact lens in the FS-LASIK group, which might have impacted corneal topography and reduced the reliability. The contact lens we used here was a silicone hydrogel lens with high oxygen transmissibility and large base curve of 8.80 mm. A Chinese population-based study found that the mean central corneal radius was 7.80 ± 0.25 mm (33). Moreover, keratorefractive surgeries mainly reshaped the central surface of anterior cornea and flattened the central curve postoperatively, especially in cases of extremely high myopia (6). Thus, this contact lens might not adhere to the cornea tightly, especially to the central optical zone postoperatively. Moreover, research showed that this contact lens may also reduce corneal edema at 24 h postoperatively (34). We assume this might also decrease the potential impact to the cornea.

## Data Availability Statement

The original contributions presented in the study are included in the article/supplementary material, further inquiries can be directed to the corresponding author/s.

## Ethics Statement

The studies involving human participants were reviewed and approved by Ethics Committee of Eye and ENT Hospital of Fudan University. The patients/participants provided their written informed consent to participate in this study.

## Author Contributions

XYZ and XTZ: study concept and design. XYZ, BQ, TH, JS, and ZC: data collection. XYZ, JZ, and PY: analysis and interpretation of data. XYZ: writing the manuscript and final approval of article. XTZ: critical revision of the manuscript. BQ, TH, and JZ: administrative, technical, and material support. All authors contributed to the article and approved the submitted version.

## Conflict of Interest

The authors declare that the research was conducted in the absence of any commercial or financial relationships that could be construed as a potential conflict of interest.

## Publisher’s Note

All claims expressed in this article are solely those of the authors and do not necessarily represent those of their affiliated organizations, or those of the publisher, the editors and the reviewers. Any product that may be evaluated in this article, or claim that may be made by its manufacturer, is not guaranteed or endorsed by the publisher.
